# Children Raised by Grandparents or Nongrandparents: Which Have Greater Odds of High Academic Performance?

**DOI:** 10.1093/geroni/igaa057.235

**Published:** 2020-12-16

**Authors:** Tamar Shovali, Melissa Bright, Kerstin Emerson

**Affiliations:** 1 Eckerd College, St Petersburg, Florida, United States; 2 University of Florida, Gainesville, Florida, United States; 3 University of Georgia, Athens, Georgia, United States

## Abstract

There is a 19-fold greater likelihood that children removed from parental care will be raised by a grandparent than any other caregiver. Theorists and practitioners highlight the importance of monitoring academic progress to understand the benefits of at-risk youth living in grandfamilies. Using a nationally representative dataset we examined academic performance for children in three caregiver (N = 814) categories: Grandparent (73.1%), Foster parent (12.7%), Other (nonfoster, nonkin/nonfoster; 14.2%), with significance testing across groups. Children were between 6-17 years with grandfamilies and foster families caring for significantly younger children compared to the “other” group. Overall, 76% of children were reported to have high academic performance in math and 79.6% had high academic performance in reading/writing. Grandparents were caring for a significantly higher proportion of non-Hispanic White children with statistically significantly higher reported academic performance in math and reading/writing compared to nongrandparents. Logistic regression model A showed for both foster parent (AOR 0.57, CI: .35-.91) and other (AOR 0.55, CI: .35-.86) caregiver groups were significantly negatively correlated with high math performance compared to grandparents. Model B showed the same statistically significant and negative relationship to reading/writing performance outcomes for foster parent (AOR 0.56, CI: .02-.35) and other (AOR 0.51, CI: .01-.32) caregivers compared to grandparents. Controlling for relevant caregiver and child variables both models suggest that children living with grandparents have 55% greater odds of high academic performance compared to children raised by nongrandparents. Findings support placement of children with grandparents. Supporting grandfamilies with appropriate social services will be reviewed.

